# Circulating endothelin-1 levels are positively associated with chronic kidney disease in women but not in men: a longitudinal study in the Vara-Skövde cohort

**DOI:** 10.1186/s12882-021-02525-5

**Published:** 2021-10-02

**Authors:** Margareta I. Hellgren, Per-Anders Jansson, Hormoz Alayar, Ulf Lindblad, Bledar Daka

**Affiliations:** 1 Department of Public Health and Community Medicine/Primary Health Care, the Sahlgrenska Academy at the University of Gothenburg, Gothenburg, Sweden; 2 The Wallenberg Laboratory, Department of Molecular and Clinical Medicine, the Sahlgrenska Academy at the University of Gothenburg, Gothenburg, Sweden

**Keywords:** Endothelin-1, Kidney disease, Gender, Epidemiology

## Abstract

**Background:**

The vasoconstricting peptide endothelin-1 (ET-1) is associated with endothelial dysfunction. The aim of this paper was to investigate whether circulating ET-1 levels predicts chronic kidney disease (CKD) in a prospective population study.

**Methods:**

In 2002–2005, 2816 participants (30–74 years) were randomly selected from two municipalities in South-Western Sweden and followed up in a representative sample of 1327 individuals after 10 years. Endothelin-1 levels were assessed at baseline. Outcome was defined as CKD stage 3 or above based on eGFR < 60 mL/min/1.73m^2^. Those 1314 participants with successful analysis of ET-1 were further analyzed using binary logistic regression.

**Results:**

At follow-up, 51 (8%) men and 47 (7,8%) women had CKD stage 3 and above. Based on levels of ET-1 the population was divided into quintiles showing that women in the highest quintile (*n* = 132) had a significantly increased risk of developing CKD during the follow up period (OR = 2.54, 95% CI:1.19–5.45, *p* = 0.02) compared with the other quintiles (1–4). The association was borderline significant after adjusted for age, current smoking, alcohol consumption, hypertension, diabetes, BMI, high- sensitive CRP and LDL-cholesterol (OR = 2.25, 95% CI:0.97–5.24, *p* = 0.06). No significant differences were observed between quintiles of ET-1 and development of CKD in men (NS).

**Conclusions:**

High levels of ET-1 are associated with development of CKD in women.

## Background

Chronic Kidney Disease (CKD) is a condition characterized by a progressive loss of kidney function over time and with a high prevalence, particularly amongst individuals with diabetes and hypertension [1–5]. The condition is regarded as a global health problem and affect 10–16% of the world’s adult population [6]. Normally, glomerular filtration rate (GFR) declines over the years and this process starts as early as 30 years of age only to accelerate after 60 years of age [6]. Furthermore, co-morbidity with cardio-vascular disease is high [7]. To prevent this deleterious development and offer proper preventive care early detection is of great importance [8].

The connection between kidney disease and endothelin-1 (ET-1) is fairly well explored [9]. Current understanding of CKD is that pathological changes, such as glomerular sclerosis, interstitial fibrosis, glomerular hypertension, cellular hypertrophy, inflammation and extracellular matrix congestion are commonly implicated in the pathophysiology of the disease [9]. These changes, to varying degree, contribute to the decline in kidney function [10–12]. Glomerular endothelium produces the main portions of ET-1 in the kidneys [13] and is referred to as a paracrine production, however, it is the serum levels that is usually available for analyses. Elevated levels of ET-1 can damage the kidneys podocytes, cause structural change and cause a decline in the barrier function [12]. Over time these actions contribute to the decline of the kidney structure and in essence the decline in kidney function. Thus, previous experimental studies indicate that ET-1 is a central component of the mechanisms behind the development of CKD [9]. In addition, levels of ET-1 have been reported to be associated with increased risk to develop diabetes, insulin resistance and coronary heart disease in women, but not in men [14–16]. However, the impact of serum levels of ET-1 on kidney disease has to the best of our knowledge never been reported before in an epidemiological context. Therefore, we set out to examine the relationship between circulating ET-1 levels and CKD in the longitudinal population-based study of the Vara-Skövde cohort.

## Methods

### Study population

**Fig. 1:**
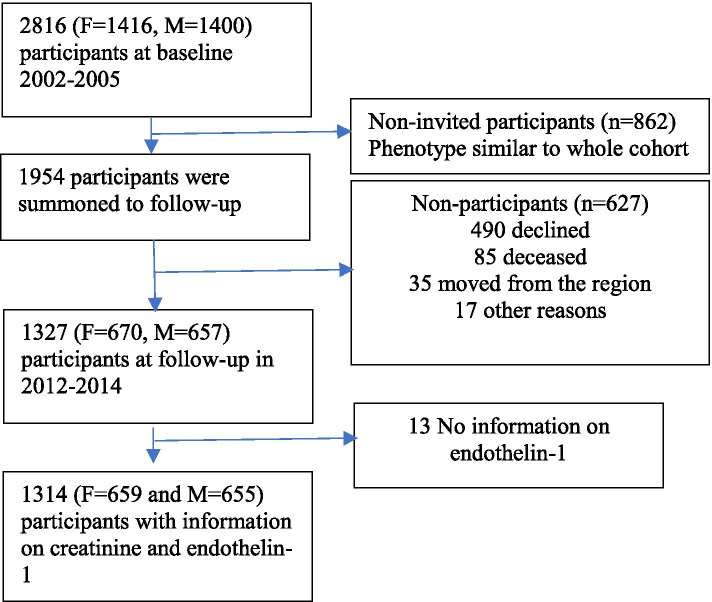
Flow-chart of the study population. M: men; W Women

In 2002–2005, 2816 participants (30–74 years) were randomly selected from two municipalities in South-Western Sweden. There was an oversampling of individuals younger than 50 years and mean age in the population was 48 years (±11 years). The cohort was followed up with similar procedures in 2012–2014 and 1327 representative participants were included in this follow-up. The mean follow-up time was 9.7 years. We excluded subjects where the analysis for ET-1 was unsuccessful (*n* = 13), leaving 1314 participants for further analyses. See flow-chart of the study (Fig. 1). The participants at follow-up were consecutively invited and were considered representative for the complete population as confirmed by participant/non-participant analyses showing no difference concerning base-line characteristics.

### Measurements, analyses and blood sampling

The details of the procedures for this cohort have been described previously [17, 18]. Briefly, all participants were carefully examined with anthropometric data, blood pressure, body weight and height, waist circumference, and sampling of fasting venous blood. Lifestyle factors, such as physical activity, current smoking, and alcohol consumption was reported through validated questionnaires. ET-1 levels in plasma were assessed at baseline using the QuantiGlo Chemiluminescent ELISA for Human ET-1. Creatinine was measured at baseline and at follow-up. Kidney function was estimated based on MDRD-4 as well as on the CKD-EPI formula for the calculation of eGFR at baseline, and again at follow-up. Analyses from the CKD-EPI formula are reported in this paper. The main outcome in this study is the incidence of CKD defined as eGFR below 60 mL/min/1.73m^2^, ie entering CKD stage 3 and above.

### Statistical methods

The study population was described using standard methods for descriptive statistics. Logistic regression analyses were computed to explore the association between quintiles of ET-1 and CKD separately with adjustments in theoretical models. Individuals with CKD (eGFR< 60 ml/min/1,73m^2^) at baseline were excluded from the multivariate analyses. Two way interaction test for gender and ET-1 showed a significant association (*p* = 0.019) and therefore the analyses were carried on separately for men and women. SPSS for Macintosh version 27 was used for data analyses.

## Results

After a mean follow-up time of 9.7 years (SD1.4), 98 cases of CKD stage 3 or higher were detected, 51 (8%) men and 47 (7,8%) in women, as compared to baseline when 8 (1.2%) men and 16 (2.4%) women had CKD stage 3 or more*.* There was a significant decline in eGFR in both men and women, − 9.5 mL/min/1.73 m^2^ (1 mL/min/1.73 m^2^ per year) and – 7.8 mL/min/1.73 m^2^ (0.8 mL/min/1.73 m^2^ per year) respectively (*p* < 0.001). CKD stage 3 and above was more common in advanced age groups, with 40% of all CKD in individuals > 70 years of age at follow-up.

### Endothelin-1 quintiles and clinical characteristics

ET-1 concentrations were slightly higher in women than in men when adjusted for age and BMI (diff = 0.14, *p* = 0.049, 95% CI:0.00–0.27). Characteristics for men and women by ET-1 quintiles are shown in Tables 1 and 2 respectively. There was a significant decline in eGFR across all quintiles at the 9.7-year follow-up (*p* < 0.001). At baseline, the mean value of HOMA-IR for individuals in the highest quintile of ET-1 was significantly higher than for other quintiles in both men and women (diff = 0.4 *p* = 0.001 95% CI:0.14–0.53).

**Table 1:** Baseline characteristics in men presented as endothelin-1 quintiles

Men	Q1 ***n*** = 131	Q2 ***n*** = 131	Q3 ***n*** = 131	Q4 ***n*** = 131	Q5 ***n*** = 131
Age (years)	50 ± 12	49 ± 12	48 ± 12	50 ± 13	50 ± 11
eGFR < 60% (n)	2% (2)	2% (3)	0.00% (0)	2% (3)	0% (0)
eGFR < 60% (n) + 9,7 yr	10% (13)	8% (10)	11% (14)	6% (7)	6% (7)
eGFR mean	89 ± 15	91 ± 17	89 ± 13	92 ± 15	91 ± 15
eGFR mean + 9.7 yr	80 ± 14	82 ± 17	80 ± 15	82 ± 13	82 ± 14
Diabetes (%)	6.1%	3.8%	3.8%	3.8%	9.2%
Diabetes (%) + 9,7 yr	13.0%	9.9%	11.5%	10.7%	15.3%
Hypertension (%)	13.7%	20.6%	13.7%	16.8%	13.0%
Hypertension (%) + 9,7 yr	29.0%	35.1%	28.2%	31.3%	29.8%
Smoking (%)	18.30%	6.90%	14.50%	13.70%	16.00%
LDL	3.3 ± 0.8	3.3 ± 0.8	3.3 ± 0.9	3.5 ± 1.0	3.5 ± 0.9
LDL + 9,7 yr	3.4 ± 1.0	3.4 ± 0.9	3.6 ± 0.9	3.6 ± 1.1	3.5 ± 0.9
WHR	0.94 ± 0.06	0.94 ± 0.06	0.94 ± 0.06	0.94 ± 0.06	0.94 ± 0.06
HOMA-IR	1.6 ± 1.1	1.5 ± 1.1	1.5 ± 0.9	1.6 ± 1.3	1.9 ± 1.4^*^
hS-CRP	1.9 ± 2.0	3.3 ± 13.5	2.7 ± 7.8	2.4 ± 4.0	1.9 ± 1.8
Low LTPA	8.40%	6.10%	9.20%	6.10%	9.20%

Means ± SD, and frequency/percentages of clinical variables between endothelin-1 quintiles. HOMA-IR: homeostatic model assessment of insulin resistance; *WHR* Waist/hip ratio, *LDL* Low-density lipoprotein, *hS-CRP* High sensitive C-reactive protein, *Low LTPA* Low leisure time physical activity

Range of quintiles [ET-1] (pg/mL) 1: *0.02–1.25*, 2: *1.26–1.97*, 3: *1.98–2.61*, 4: *2.62–3.35*, 5: *3.36–6.24*

* = *P*-value < 0.05

**Table 2:** Baseline characteristics in women presented as endothelin-1 quintiles

Women	Q1 ***n*** = 131	Q2 ***n*** = 132	Q3 ***n*** = 132	Q4 ***n*** = 132	Q5 ***n*** = 132
Age (years)	49 ± 11	48 ± 11	47 ± 12	48 ± 11	49 ± 12
eGFR < 60% (n)	2% (3)	1% (1)	5% (7)	3% (4)	1% (1)
eGFR < 60% (n) + 9,7 yr	9% (11)	6% (8)	4% (5)	5% (6)	13% (16)*
eGFR mean	88 ± 13	89 ± 12	89 ± 16	88 ± 13	88 ± 14
eGFR mean + 9.7 yr	79 ± 14	81 ± 14	82 ± 16	80 ± 13	79 ± 15
Diabetes (%)	3.1%	3.0%	5.3%	4.5%	4.5%
Diabetes (%) + 9,7 yr	8.4%	3.8%	8.4%	12.1%	13.6%
Hypertension (%)	11.5%	10.6%	18.9%	15.2%	15.2%
Hypertension (%) + 9,7 yr	21.4%	25.0%	29.5%	23.5%	31.1%
Smoking (%)	16.8%	18.9%	13.6%	15.9%	12.1%
LDL	3.1 ± 0.8	3.1 ± 1.0	3.0 ± 0.8	3.3 ± 1.0	3.3 ± 0.9
LDL + 9,7 yr	3.5 ± 1.0	3.5 ± 0.9	3.3 ± 0.9	3.5 ± 0.9	3.5 ± 0.9
WHR	0.8 ± 0.08	0.8 ± 0.08	0.8 ± 0.08	0.8 ± 0.07	0.8 ± 0.08
HOMA-IR	1.4 ± 1.0	1.3 ± 1.0	1.4 ± 0.9	1.4 ± 1.2	1.9 ± 3.5*
hS-CRP	2.6 ± 3.9	2.4 ± 5.2	3.0 ± 8.3	2.5 ± 2.8	3.4 ± 4.2
Low LTPA	6.9%	4.5%	3.0%	6.8%	3.8%

Means ± SD, and frequency/percentages of clinical variables between endothelin-1 quintiles. HOMA-IR: homeostatic model assessment of insulin resistance; *WHR* Waist/hip ratio, *LDL* Low-density lipoprotein, *hS-CRP* High sensitive C-reactive protein, *Low LTPA* Low leisure time physical activity

Range of quintiles [ET-1] (pg/mL) 1: 0.02–1.39, 2: 1.40–2.04, 3: 2.05–2.71, 4: 2.72–3.68, 5: 3.69–6.10

* = *P*-value < 0.05

### Quintiles of endothelin-1 and association with CKD stage 3 or higher

Baseline circulating ET-1 levels in the highest quintile, as compared to the quintile three, in women significantly increased the risk of reaching CKD stage 3 or higher during the follow up period, whereas this effect was not observed in men as shown in Table 3. This predictive value of ET-1 for incident CKD stage 3 or higher in women was borderline significant (*p* = 0.06) after adjustments for age, body mass index (BMI), exercise, smoking, alcohol, hypertension, diabetes, high-sensitive CRP and LDL-cholesterol. (Table 3)*.* Furthermore, adjustments for medication with statins, angiotensin converting enzyme inhibitors and angiotensin receptor blockers didn’t change the results significantly (OR 2.19, 95% CI: 0.94–5.12, *P* = 0.06).

**Table 3:** Association between circulating endothelin-1 presented as quintiles at baseline and development of chronic kidney disease over a 9.7 years observation period

**Male sex (CKD*n*** **= 38)**	**OR**	**CI**	***P-*value**
Model 1	0.47	0.18–1.20	0.113
Model 2: Model 1 + Confounders	0.46	0.17–1.27	0.135
Model 3: Model 2 + Pathways	0.43	0.14–1.25	0.121
**Female sex (CKD*n*** **= 44)**	**OR**	**CI**	***P-*value**
Model 1	2.54	1.19–5.45	0.017
Model 2: Model 1 + Confounders	2.63	1.20–5.74	0.016
Model 3: Model 2 + Pathways	2.25	0.97–5.24	0.060

Model 1: adjusted for estimated glomerular filtration rate at baseline and age

Model 2: adjusted for model 1 + alcohol consumption, smoking

Model 3: adjusted for model 2 + hypertension, diabetes, high sensitive C-reactive protein, low density lipoprotein and body mass index

*CKD* Chronic kidney disease

Interestingly, when using the MDRD formula ET-1 was significantly predictive for CKD stage 3 even after full adjustments (OR 2.48, CI: 1.19–5.18, *P* = 0.02).

**Fig. 2:**
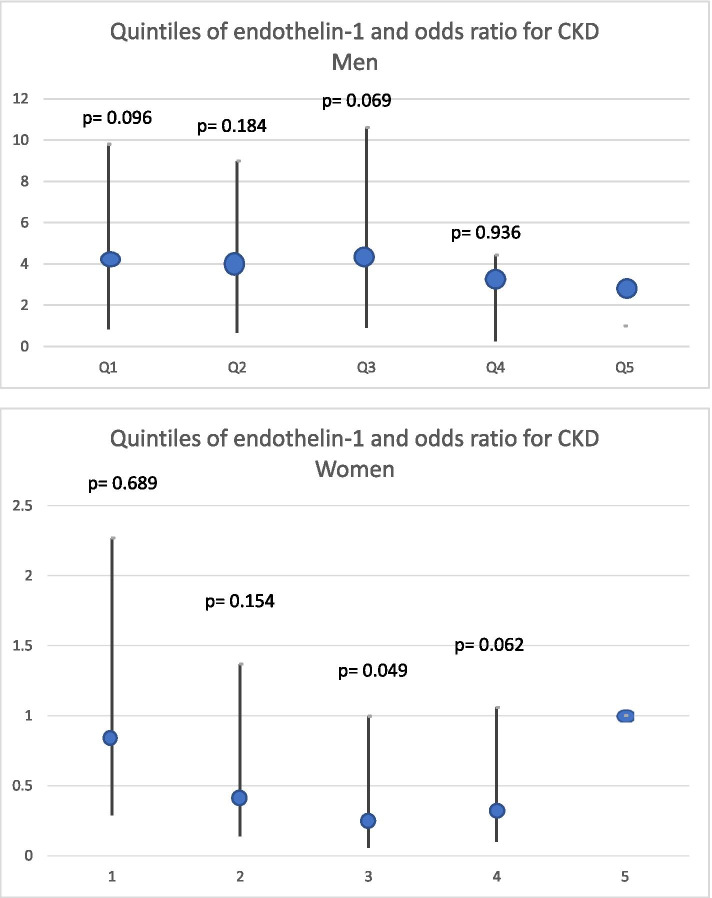
The association between quintiles of endothelin-1 and the odds ratio to develop chronic kidney disease stage 3 (eGFR < 60 mL/min/1.73 m^2^) in men and women. A logistic regression model was used and OR presented for associations between quintiles of circulating endothelin-1 concentrations at baseline and incident CKD over a 9.7 years observation period with the highest quintile as reference. Adjustments were made for age, eGFR at baseline, body mass index and follow-up time. OR: Odds ratio; CKD: chronic kidney disease; eGFR: estimated glomerular filtration rate; CI: confidence interval min and max indicated

Quintiles of ET-1 and OR for the CKD incidence is presented in Fig. 2 showing a j-shaped association in women where high levels of ET-1 showed a significantly higher risk to develop CKD than the third quintile, showing lower odds for all other quintiles. The curve also indicated a non-significantly increased risk even for those in the lowest quintile.

## Discussion

In this longitudinal observational study, we could show that a high level of circulating ET-1 at baseline was associated with progression to CKD stage 3 or higher at follow-up after 10 years in women, but not in men. This association was independent of age and eGFR at base-line but was attenuated by BMI. These findings suggest that ET-1 may be a predictor of CKD in women.

It has been established on population basis that circulating ET-1 could help predict chronic heart disease and diabetes, but repeatedly that it lacks a significant association to hypertension [10]. These findings suggest that endothelial dysfunction per se might influence the deterioration of kidney function independently of hypertension. Generally, in CKD we can observe pathological changes where ET-1 has a central and well mapped physiological role, a mechanism that may precede a detectable decrease in eGFR and albuminuria by causing increased glomerular hypertension [10], cellular hypertrophy and inflammation [19]. Speculatively, discrete endothelial dysfunction in a younger population might precede other observable risk factors such as decreased eGFR or albumin leakage, which are surrogates for disease progression, and ET-1 could act as a risk marker for metabolic disturbances connected to endothelial dysfunction. CKD is a large contributor to health decline in the aging population and is usually detected late in the disease progression. Early detection would be of great benefit to the patient and preventive care could be initiated much sooner if patients at risk could be identified at an early stage. The Vara-Skövde cohort in this study is still considered young and many of the participants are still at an age where the disease has not yet emerged. Our paper may thus add to the understanding of the role of ET-1 in a longitudinal population perspective, but further studies are warranted.

In the analyses we used both the MDRD formula and the CKD-EPI formula. There were some differences showing significant results with the MDRD formula that could not be repeated using the CKD-EPI formula. However, the tendency was the same and we suspect that border-line significance using the CKD-EPI formula may be due to a lack of power.

This study also indicates considerable sex differences where women seem to be more vulnerable to the deleterious effect of ET-1. Sex differences are observed in previous studies in the development of cardiovascular disease, possibly due to differences regarding ET-1 and the regulation of vascular tone and receptor response [20, 21]. Data also suggest that ET-1 production in the vascular endothelium is attenuated in the presence of estrogens and progesterone [22]. Moreover, to stress the complexity of ET-1, these mechanistic studies focus more on the sensitivity for ET-1 in men and even speculate that ET-1 may be one of the reasons for the higher risk for CVD events in men as compared to premenopausal women [23], while, in our population-based study, we report levels of ET-1 to be of higher importance in women than in men. A part from the studies from the Skaraborg project there are very few population-based studies and none, to the best of our knowledge, reporting differences between the sexes [24, 25]. In addition, previous results from this cohort has shown that serum concentrations of ET-1 can predict development of diabetes, high insulin resistance as well as an increased CHD risk in women but not in men consistent with findings in this study [14, 15].

### Strengths and limitations

The study has a long follow-up period and a high participation rate. The age distribution of the population has both advantages and disadvantages. A main disadvantage being that the younger age of the studied population and the use of eGFR to define CKD underestimates the presence of damage to the kidneys. The method cannot differentiate between individuals that still have a reasonably high GFR but various levels of structural damage to the glomerular units.

The OR curve is slightly J shaped, however not significant, indicating possible power issues that could be remedied by a larger study population. Anyhow, it might reflect that the association between ET-1 levels and CKD-risk is not linear. It may be that the best levels of ET-1 are those in the middle and both high and low levels suggest a dysfunction in the endothelium and thus increased risk for CKD and CVD events. Also, the incidence of CKD in men is limited in this study and the lack of association may be a power problem and a lack of association between ET-1 and CKD in men cannot be definitely outrolled.

One other consideration is that the ET-1 measured in this study is circulating serum concentrations and not the paracrine production in the kidney. However, in pragmatic day to day clinical practice we have no access to the paracrine measurements without advanced and invasive methods. Benefits of studying circulating levels, should they show clinical significance, present a method more readily accessible to clinicians and would be easier to implement in daily clinical routine.

## Conclusions

In conclusion, high circulating ET-1 levels were associated, even if not significantly, with development of CKD stage 3 or higher in women but not in men. Sex differences, also observed in previous studies, have been confirmed in this study, indicating that women might be more vulnerable to increased levels of ET-1 than men.

## Data Availability

The data analysed during the current study are available from the corresponding author on reasonable request.

## Abbreviations

ET-1: Endothelin 1
CKD: Chronic Kidney Disease
eGFR: Estimated glomerular filtration rate
CRP: C-reactive protein
OR: Odds ratio
LDL: Low Density Lipoprotein
CI: Confidence Interval
CVD: Cardio Vascular Disease
